# 3D confinement alters smooth muscle cell responses to chemical and mechanical cues

**DOI:** 10.1063/5.0225569

**Published:** 2024-10-25

**Authors:** Farnaz Hemmati, Ayuba Akinpelu, Daniel Chinedu Nweze, Panagiotis Mistriotis

**Affiliations:** Department of Chemical Engineering, Auburn University, Auburn, Alabama 36849, USA

## Abstract

Smooth muscle cell (SMC) phenotypic switching is a hallmark of many vascular diseases. Although prior work has established that chemical and mechanical cues contribute to SMC phenotypic switching, the impact of three-dimensional (3D) confinement on this process remains elusive. Yet, *in vivo*, arterial SMCs reside within confined environments. In this study, we designed a microfluidic assay to investigate the interplay between 3D confinement and different environmental stimuli in SMC function. Our results show that tightly, but not moderately, confined SMCs acquire a contractile phenotype when exposed to collagen I. Elevated compressive forces induced by hydrostatic pressure abolish this upregulation of the contractile phenotype and compromise SMC survival, particularly in tightly confined spaces. Transforming growth factor beta 1, which promotes the contractile state in moderate confinement, fails to enhance the contractility of tightly confined cells. Fibronectin and engagement of cadherin 2 suppress the contractile phenotype of SMCs regardless of the degree of confinement. In contrast, homophilic engagement of cadherin 11 upregulates SMC-specific genes and enhances contractility in both moderately and tightly confined cells. Overall, our work introduces 3D confinement as a regulator of SMC phenotypic responses to chemical and mechanical signals.

## INTRODUCTION

Malfunction of vascular smooth muscle cells (vSMCs) is one of the primary drivers of vascular diseases, including atherosclerosis, aortic aneurysm, and hypertension.^1–3^ In large arteries, SMCs primarily reside in the tunica media, and their main role is to control vascular tone by contracting or relaxing. Mounting evidence suggests that vSMCs exhibit a remarkable phenotypic plasticity. While most arterial SMCs are quiescent and exhibit a contractile phenotype, the development of various vascular diseases involves the transition of SMCs to other phenotypes.^1–3^ This phenotypic switching entails the downregulation of contractile markers and the increased expression of proteins associated with other cell types,^4^ including macrophages^5,6^ and osteoblasts.^7,8^ Additionally, contractile SMCs often undergo dedifferentiation into a synthetic phenotype, marked by the loss of contractile proteins and a substantial increase in proliferation, migration, and extracellular matrix (ECM) remodeling.^9,10^ This remodeling, driven by the excessive secretion of ECM proteins and matrix metalloproteinases, can lead to adverse mechanical, functional, and structural alterations in the vascular wall that contribute to disease progression.^11^

The phenotype of vSMCs is largely controlled by chemical and mechanical cues impinged upon cells by their local environment.^9,12^ For several decades, it has been recognized that elastin forms fenestrated sheets within the arterial wall, known as lamellae, between which SMCs and other ECM molecules reside.^13–15^ Analysis of 3D geometry of individual arterial SMCs has shown that these cells are confined *in vivo*, with widths ranging from 2 to 7 *μ*m, heights from 3 to 10 *μ*m, and lengths from 30 to 100 *μ*m.^16,17^ Although 3D confinement is a (patho)physiologically relevant cue that controls cell signaling and phenotype,^18,19^ its impact on the regulation of SMC behavior has been overlooked because most experiments involving SMCs are carried out on planar surfaces or in 3D environments, which do not allow fine-tuning of the degree of confinement independent of other physical parameters. To address this gap in knowledge, we integrated microfluidics with photopatterning to create high-throughput, polydimethylsiloxane (PDMS)-based devices that enable us to tune the degree of confinement, and support prolonged culture of vSMCs in environments that mimic the 3D space where vSMCs reside. These devices represent a significant advancement over existing confinement assays, such as a) micropatterned lines, two-dimensional (2D) micropatterned substrates, or uni-axial compression, which fail to fully confine cells in all three dimensions,^20^ or b) microniches, which provide moderate confinement because their size exceeds the average cell diameter.^21,22^ Moreover, although the use of light-based chemistries allows the generation of channels within hydrogels that induce spatial restrictions on cells, these methodologies are low-throughput.^23^ Using our assay, we investigated the interplay between confinement and different mechanical or chemical signals in the regulation of SMC behavior. Our findings demonstrate the pivotal role of 3D spatial constraints in shaping how SMCs respond to various stimuli.

## RESULTS

### Prolonged SMC culture in tightly confined microchannels coated with collagen I suppresses cell division, cell migration, and cell viability

**FIG. 1.**
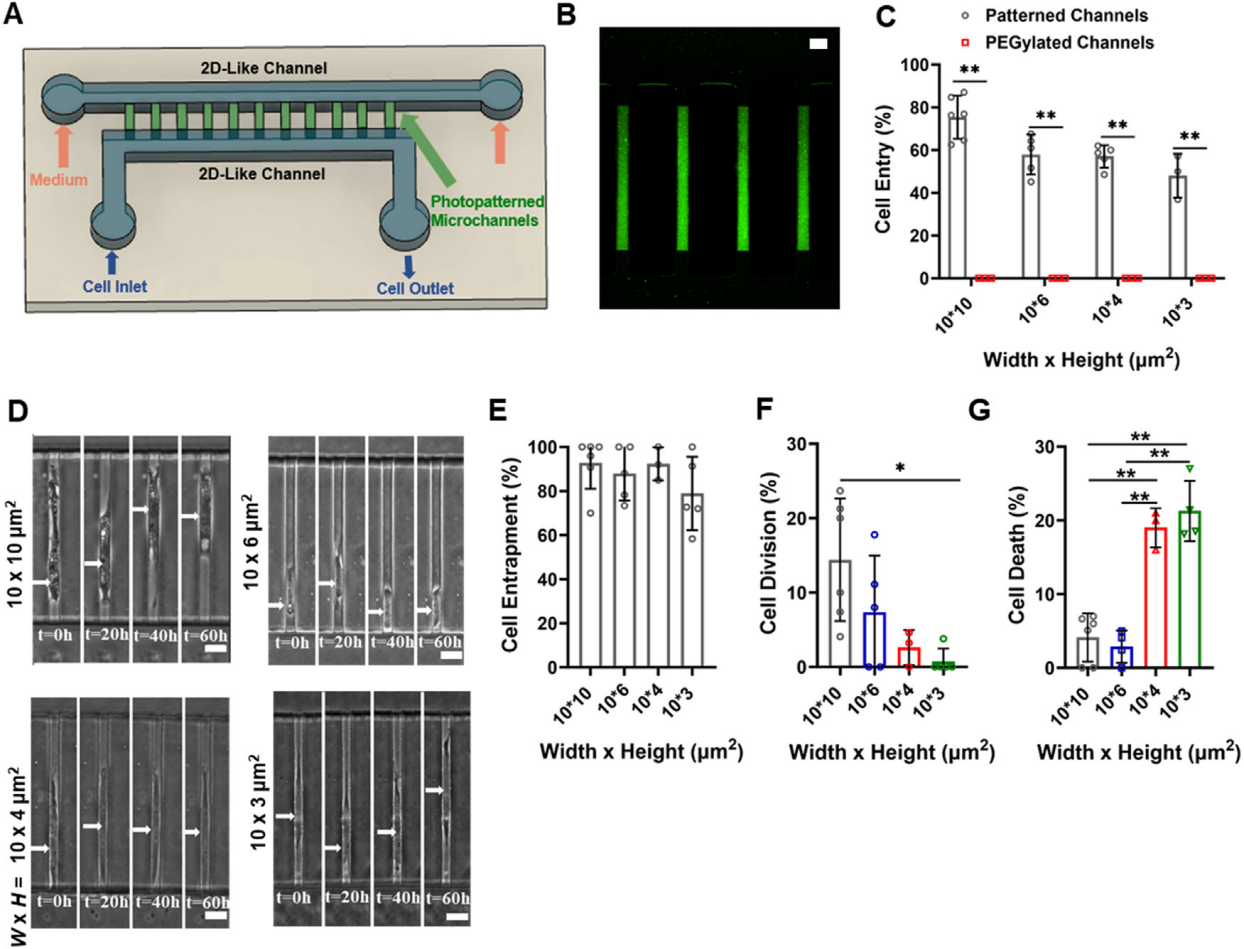
Successful SMC entrapment in confined microchannels. (a) Schematic representation of the photopatterned microfluidic device used in this study. (b) Representative confocal image showing the bottom surface of 10 *μ*m channels coated with collagen I-FITC (20 *μ*g/mL). Scale bar: 20 *μ*m. (c) Percentage of SMC entry into 10-, 6-, 4-, and 3-*μ*m collagen-I coated or PEGylated channels in response to a chemotactic gradient. Cell entry was evaluated 2h after cell seeding. At least 45 cells analyzed per experiment; at least three independent experiments. Data are mean ± S.D. ^**^ p < 0.01. Statistical comparisons are made using two-way ANOVA. (d) Image sequence showing SMC entrapment in 10-, 6-, 4-, and 3-*μ*m channels coated with collagen I for 60h. The white arrows indicate cell position. Scale bar: 20 *μ*m. (e) The percentage of SMC entrapment in 10-, 6-, 4-, and 3-*μ*m channels coated with collagen I after 18 h. At least 45 cells analyzed per experiment; at least three independent experiments. Data are mean ± S.D. Statistical comparisons are made using one-way ANOVA. (f) Percentage of SMCs that divide inside 10-, 6-, 4-, and 3-*μ*m channels coated with collagen I over the course of 18 hours. At least 45 cells analyzed per experiment; at least 3 independent experiments. Data are mean ± S.D. ^*^p < 0.05. Statistical comparisons are made using one-way ANOVA. (G) Percentage of SMCs that die inside 10-, 6-, 4-, and 3-*μ*m coated with collagen I after 3 days of entrapment. At least 45 cells analyzed per experiment; at least 3 independent experiments. Data are mean ± S.D. ^**^p < 0.01. Statistical comparisons are made using one-way ANOVA.

Because arterial SMCs are confined by ECM proteins and neighboring cells,^16,17^ we examined the impact of 3D confinement on SMC growth, survival, migration, gene expression, and contractility. We designed a polydimethylsiloxane (PDMS)-based microchannel assay that enabled cell entrapment in confining microchannels with dimensions that mimic the 3D space in which arterial SMCs reside^24^ [Fig. 1(a)]. These microchannels had fixed (*L*)ength (200 *μ*m) and (*W*)idth (10 *μ*m) but varying (*H*)eights (3–10 *μ*m), allowing us to monitor cell behavior in moderate (10 and 6 *μ*m) or tight confinement (4 and 3 *μ*m). Our devices also featured two larger 2D-like channels perpendicular to microchannels, acting as reservoirs for cells and cell culture medium [Fig. 1(a)]. Photopatterning was employed to selectively coat microchannels with collagen I [Figs. 1(a) and 1(b)], the most abundant collagen in the human aorta, constituting ∼60%–70% of the total collagen.^25^ The remaining parts of the device were treated with an anti-fouling layer composed of poly-L-lysine (PLL) and methoxy PEG (mPEG)–succinimidyl valerate (SVA) to inhibit cell adhesion.

Next, human aortic SMCs were introduced into the cell inlet [Fig. 1(a)] and seeded in the lowermost 2D-like channel adjacent to the entrances of photopatterned microchannels using pressure-driven flow. Cells were stimulated to enter these channels by a chemoattractant [5% (v/v) growth medium] placed in the uppermost 2D-like channel. ∼50%–70% of the SMCs entered the photopatterned microchannels within 2 h post-seeding, whereas no cellular ingress was noted in PEGylated microchannels (i.e., microchannels treated only with PLL-mPEG-SVA) [Fig. 1(c)]. Importantly, ∼80%–90% of the cells that entered the photopatterned microchannels remained entrapped in these environments for at least 18 h [Figs. 1(d) and 1(e)]. This result was independent of microchannel dimensions [Figs. 1(d) and 1(e)].

After successfully entrapping cells within collagen I-coated microchannels, we examined how long-term confinement affected SMC division, migration, and viability. As confinement levels increased, we observed a marked reduction in the proportion of SMCs undergoing division within 18 h [Fig. 1(f)]. This effect was most pronounced in 3- or 4-*μ*m channels, where cell proliferation came to a halt [Fig. 1(f)]. Reduced cell division in response to increased confinement has also been reported by others, albeit with different cell types.^24,26–28^ Given that accumulation of YAP1 in the nucleus promotes proliferation and the synthetic phenotype of SMCs,^29,30^ we examined its localization in moderate and tight confinement. Consistent with our proliferation data, we found that YAP1 remained predominantly nuclear in moderate confinement, whereas it exited the nucleus under tight confinement [supplementary material, Figs. 1(a) and 1(b)]. Of note, this confinement-induced cytoplasmic accumulation of YAP1 was not an artifact of immunocytochemistry, as the nuclear-to-cytoplasmic ratio of the nuclear marker Histone H3 remained unchanged between moderate and tight confinement conditions [supplementary material, Fig. 1(c)]. Furthermore, tight confinement suppressed SMC migration speed [supplementary material, Fig. 1(d)], while also triggering cell death in ∼15%–20% of SMCs, as observed using live–dead staining [Fig. 1(g)].

### Aortic SMCs acquire a contractile phenotype in tightly confined microchannels coated with collagen I

**FIG. 2.**
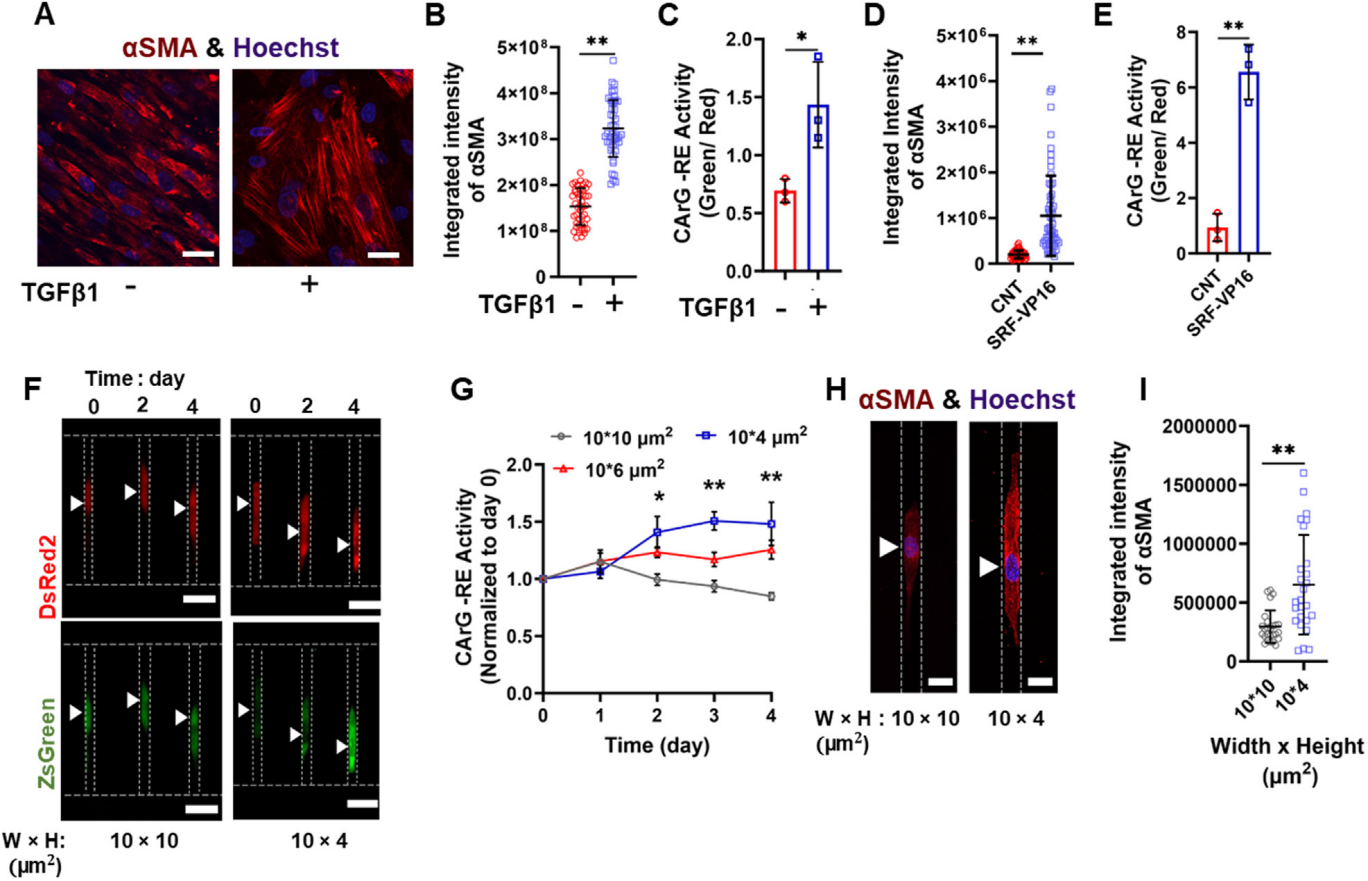
Long-term 3D confinement regulates SMC gene expression. (a) Representative confocal images showing αSMA expression in SMCs cultured on glass surfaces in the presence or absence of TGFβ-1 (2 ng/mL). Scale bar: 100 *μ*m. (b) Quantification of (a). At least 16 cells analyzed per experiment; 3 independent experiments. Data are mean ± S.D. ^**^p < 0.01. Statistical comparisons are made using t-test. (c) Quantification of CArG-RE activity in SMCs cultured on glass surfaces in the presence or absence of TGF-β1 (2 ng/mL). 10 images analyzed per experiment; 3 independent experiments. Data are mean ± S.E.M. ^*^p < 0.05. Statistical comparisons are made using t-test. (d) Quantification of αSMA levels in SMCs transduced with lentivirus expressing puromycin resistance (Puro) gene (CNT) or constitutively active SRF and Puro (SRF-VP16). At least 20 cells analyzed per experiment; 3 independent experiments. Data are mean ± S.D. ^**^p < 0.01. Statistical comparisons are made using t-test. (e) Quantification of CArG-RE activity in CNT SMCs or SMCs expressing SRF-VP16. 10 images analyzed per experiment; 3 independent experiments. Data are mean ± S.E.M. ^**^p < 0.01. Statistical comparisons are made using t*-*test. (f) Image sequence showing CArG-RE activity in SMCs entrapped in 10- or 4-*μ*m channels coated with collagen I. The white arrowheads indicate cell position. Scale bar: 20 *μ*m. (g) CArG-RE activity (normalized to day 0) as a function of time in SMCs entrapped in 10-, 6-, or 4- μm channels coated with collagen I. At least 12 cells analyzed per experiment; at least 3 independent experiments. Data are mean ± S.E.M. ^*^p < 0.05, ^**^ p < 0.01 between 10- and 4-*μ*m channels. Statistical comparisons are made by using two-way ANOVA. (h) Representative confocal images showing αSMA levels in SMCs entrapped in 10- or 4-*μ*m channels coated with collagen I. The arrowheads indicate nuclear position. Scale bar: 10 *μ*m. (i) Quantification of (h). 10 cells analyzed per experiment; 3 independent experiments. Data are mean ± S.D. ^**^ p < 0.01. Statistical comparisons are made using t-test.

The reduced SMC proliferation, migration, and YAP1 nuclear accumulation in tightly confined channels coated with collagen I prompted us to hypothesize that increased spatial restrictions promoted the contractile phenotype of SMCs. To test this, we employed a lentiviral dual promoter (LVDP) vector that allowed us to monitor contractile gene expression in single cells, *in real-time*, and independent of virus titer.^31–33^ The LVDP vector contained two independent gene cassettes. In the first cassette, the fluorescent reporter DsRED2 was expressed under a constitutively active promoter (human phosphoglycerate kinase) and was indicative of the transduction efficiency (viral copies/cell), while in the second cassette, the reporter ZsGreen was driven by the serum response factor (SRF) binding motif [aka CArG-Response Element (RE)], which is present in the promoter region of almost all SMC-specific contractile genes, including Myh11, desmin, SM22α, alpha smooth muscle actin (αSMA), smoothelin, and caldesmon, and its activation is a marker of differentiation toward SMCs.^9,34–40^ By calculating the green-to-red fluorescence intensity (GFI/RFI) ratio, we monitored the dynamics of CArG-RE activation, regardless of transduction efficiency. We validated that the CArG-RE live-cell reporter worked as expected by measuring CArG-RE activity on 2D surfaces in response to two inducers of the contractile phenotype, namely, a constitutively active SRF (SRF-VP16) and transforming growth factor beta 1 (TGF-β1; 2 ng/mL).^9,40–42^ Both SRF-VP16 and TGF-β1 treatment increased, as expected, the expression of the smooth muscle cell marker alpha smooth muscle actin (αSMA) and markedly upregulated the CArG-RE activity [Figs. 2(a)–2(e)].

Next we quantified the activity of CArG-RE in collagen I-coated microchannels. Fluorescence imaging revealed that with an increase in days of entrapment (2–4 days), CArG-RE activity increased in 4-*μ*m but not in 10-*μ*m channels [Figs. 2(f) and 2(g)]. A modest upregulation of SRF-dependent gene expression was also noted in 6-μm channels. However, this activation was not significantly different from that observed in 4- or 10-*μ*m channels [Fig. 2(g)].

**FIG. 3.**
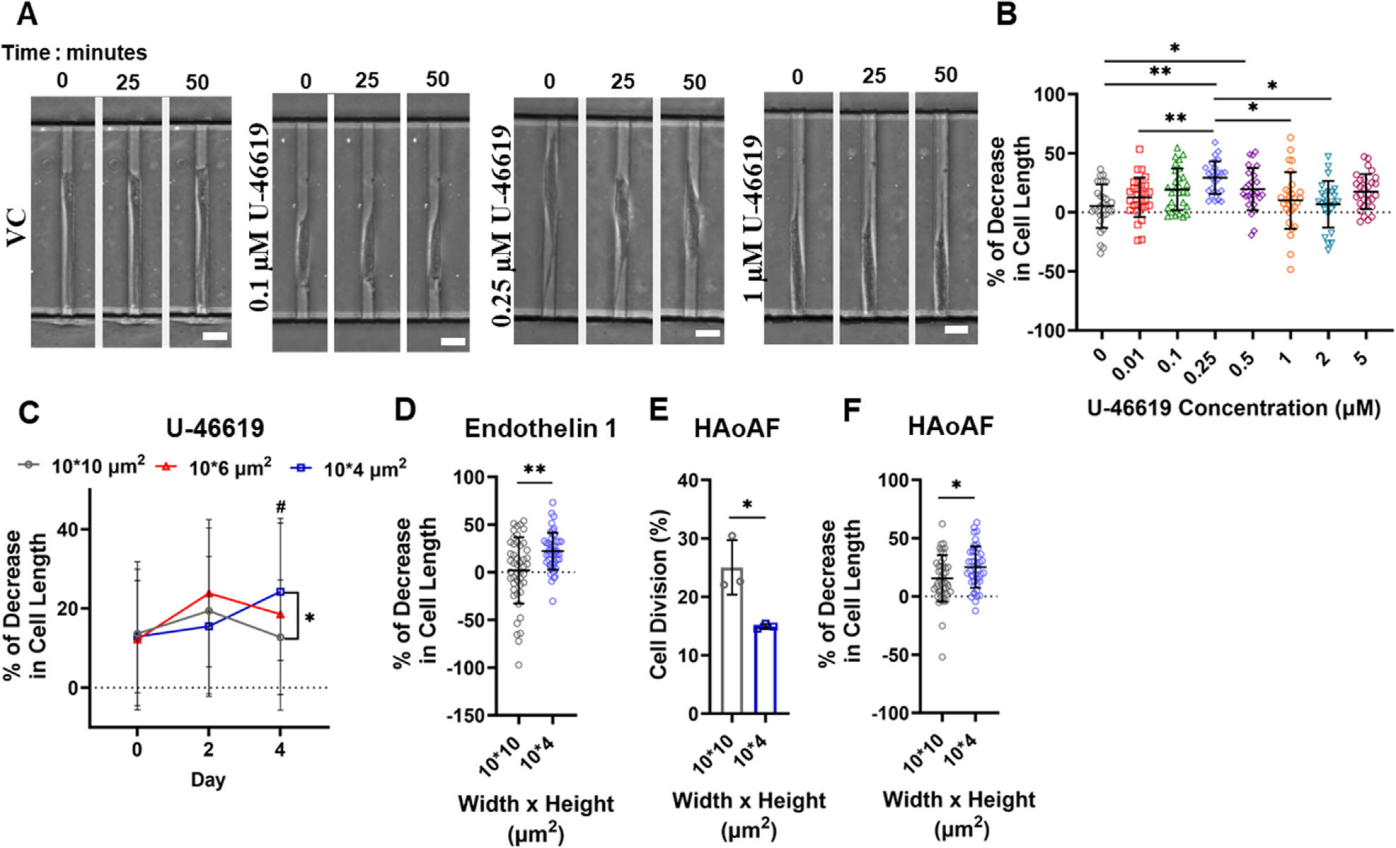
Long-term 3D confinement regulates SMC contractility. (a) Image sequence showing SMC contraction in response to different concentrations of the vasoconstrictor U-46619 after 4 days of entrapment in 10-*μ*m channels coated with collagen I. Scale bar: 10 *μ*m. (b) Percent decrease in SMC length in response to different concentrations of the vasoconstrictor U-46619 after 4 days of entrapment in 10-μm channels coated with collagen I. At least 25 cells analyzed per experiment; at least 3 independent experiments. Data are mean ± S.D. ^*^p < 0.05, ^**^p < 0.01. Statistical comparisons are made using one-way ANOVA. (c) Percent decrease in SMC length in response to 0.25 *μ*M U-46619 in 10-, 6-, οr 4-*μ*m channels coated with collagen I on days 0, 2, and 4 of entrapment. At least 12 cells analyzed per experiment; at least 3 independent experiments. Data are mean ± S.D. ^*^p < 0.05 for comparison between 10- and 4-*μ*m channels on day 4. # p < 0.05 for comparison between days 0 and 4 for 4-*μ*m channels. There was no significant difference between days 0, 2, and 4 for 6-*μ*m or 10-*μ*m channels. Statistical comparisons are made using two-way ANOVA. (d) Percent decrease in SMC length in response to 25 nM endothelin 1 in 10- οr 4-*μ*m channels coated with collagen I on day 4 of entrapment. At least 12 cells analyzed per experiment, 3 independent experiments. Data are mean ± S.D. ^**^p < 0.01. Statistical comparisons are made using t-test. (e) Percentage of HAoAF that divide inside 10- or 4- *μ*m channels coated with collagen I over the course of 18 h. At least 45 cells analyzed per experiment; 3 independent experiments. Data are mean ± S.D. ^*^p < 0.05. Statistical comparisons are made using t-test. (f) Percent decrease in HAoAF length in response to 0.25 *μ*M U-46619 in 10- οr 4-*μ*m channels coated with collagen I on day 4 of entrapment. At least 12 cells analyzed per experiment; 3 independent experiments. Data are mean ± S.D. ^*^p < 0.05. Statistical comparisons are made using t-test.

The enhanced SRF activity in 4-*μ*m channels coated with collagen I suggested that tight confinement increased the expression of SMC-specific genes. This was further confirmed through αSMA immunocytochemistry and subsequent confocal imaging [Figs. 2(h) and 2(i)]. Furthermore, we assessed the contractile function of SMCs in confining microchannels by quantifying the percent decrease in cell length in response to U-46619, a thromboxane A2 receptor agonist that stimulates contraction. Dose–response and time-dependent analyses indicated that the maximum contraction of moderately confined SMCs occurred 50 min after adding 0.25 *μ*M of U-46619 [Figs. 3(a) and 3(b)]. Under these optimized experimental conditions, we observed a progressively increasing contractile response to U-46619 in 4-*μ*m channels over time, reaching ∼25% reduction in SMC length on day 4 [Fig. 3(c)]. In comparison, SMCs cultured in 10- or 6-*μ*m channels displayed a relatively steady contractile behavior throughout the duration of the experiment, with a maximal decrease in cell length not exceeding ∼18% on day 4 [Fig. 3(c)]. Importantly, a simple media change, without the addition of U-46619, did not cause a reduction in SMC length, suggesting that these effects were specifically induced by the contractile agent [supplementary material, Fig. 1(e)]. U-46619 also triggered a decrease in other morphological parameters associated with contraction, such as cell area and aspect ratio [supplementary material, Figs. 1(f) and 1(g)]. In line with data shown in Fig. 3(c), these effects were more pronounced under tight confinement compared to moderate confinement after 4 days of entrapment [supplementary material, Figs. 1(f) and 1(g)]. Furthermore, we measured SMC length in the presence of another vasoconstrictor, endothelin 1, and observed increased contractile responses under tight confinement, similar to those induced by U-46619 [Fig. 3(d)]. It is worth noting that the mechanisms by which endothelin 1 and U-46619 induce contraction differ since endothelin 1 binds to endothelin receptors while U-46619 to thromboxane receptors. In sum, we have demonstrated that in the presence of collagen I, tight confinement upregulates αSMA and CArG-RE activity and enhances SMC contractility, as shown using two distinct vasoconstrictors. These data indicate that this microenvironment promotes the contractile phenotype of SMCs.

Arteries also contain other cell types that may be affected by confinement, such as fibroblasts, which reside within a collagen I-rich environment in the tunica adventitia.^43^ Using human aortic adventitial fibroblasts (HAoAF), we found that collagen I-coated 4-*μ*m channels suppressed the proliferation of these cells and increased their contractile response to U-46619 compared to 10-*μ*m channels [Figs. 3(e) and 3(f)]. Thus, in addition to promoting the contractile phenotype of SMCs, tightly confined spaces rich in collagen I may also trigger the differentiation of fibroblasts into myofibroblasts.

### Cell–ECM and cell–cell interactions control SMC function in confinement

In addition to collagen type I, the tunica media of arteries consist of other ECM molecules, including fibronectin and laminin.^15^ To test how different ECM proteins affected SMC behavior within confined environments, we photopatterned 4- and 10-*μ*m channels with fibronectin or laminin and compared their effects to those elicited by collagen I. While coating the microchannel surfaces with fibronectin [supplementary material, Fig. 2(a)] facilitated SMC entry into these channels [supplementary material, Fig. 2(b)], laminin deposition inhibited entry presumably due to the reduced adhesion of SMCs to laminin compared to fibronectin or collagen I, as demonstrated by monitoring cell attachment on rectangular patterns coated with these ECM molecules [supplementary material, Fig. 2(c)]. This inhibition of cell entry prevented us from entrapping SMCs within laminin-coated microchannels and consequently, our focus was redirected toward comparing the effects of collagen I and fibronectin.

**FIG. 4.**
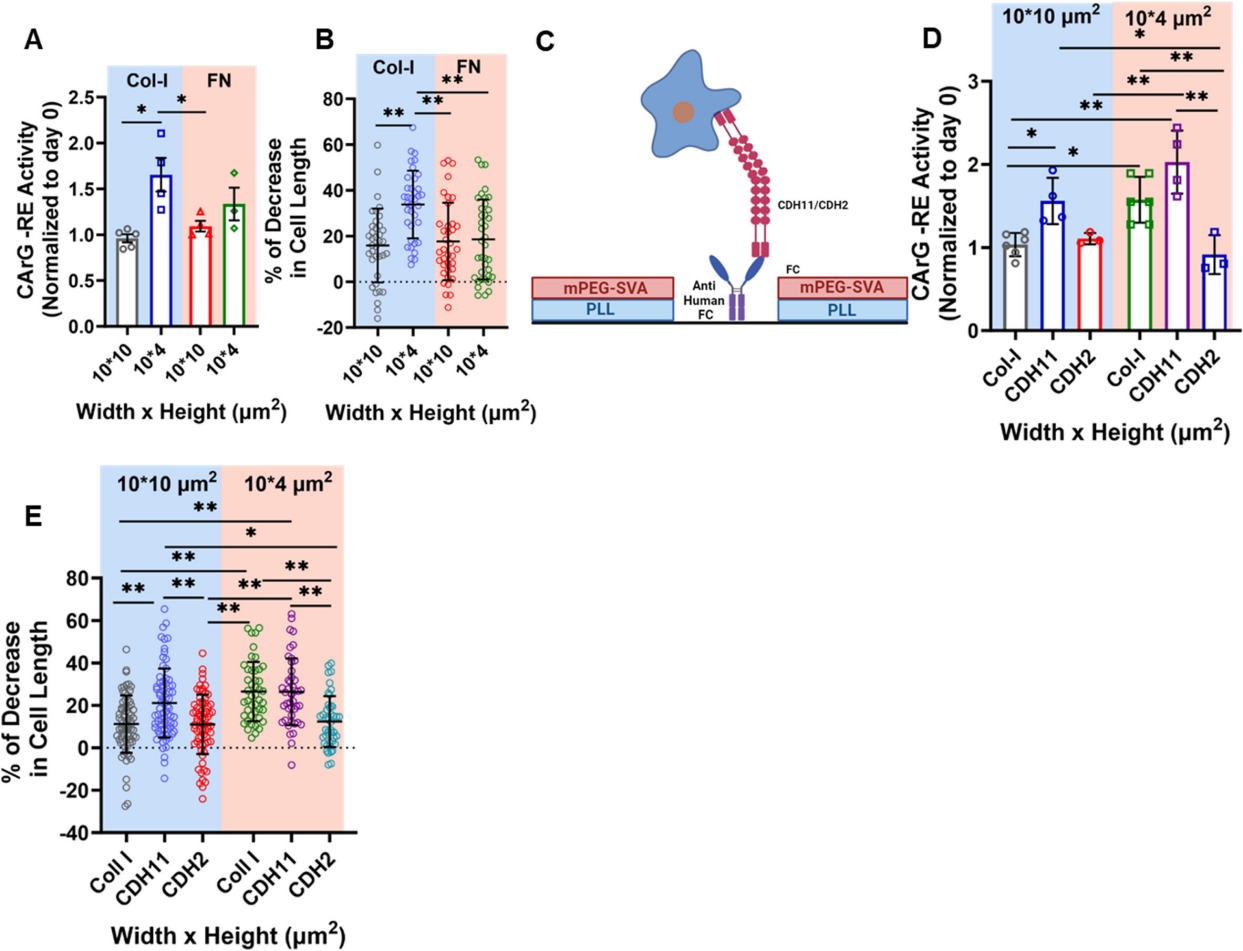
Cell–ECM and cell–cell interactions regulate SMC gene expression and contractility in confinement. (a) Quantification of CArG-RE activity on day 4 (normalized to day 0) in SMCs entrapped in 10- or 4-*μ*m channels coated with collagen I or fibronectin. At least 12 cells analyzed per experiment; at least 3 independent experiments. Data are mean ± S.E.M. ^*^p < 0.05. No statistically significant difference was observed between 4-*μ*m channel with fibronectin and 4-*μ*m channel with collagen I and between 4-*μ*m channel with fibronectin and 10-*μ*m channel with either fibronectin or collagen-I. Statistical comparisons are made using two-way ANOVA. (b) Percent decrease in SMC length in response to 0.25 *μ*M U-46619 in 10- or 4-*μ*m microchannels coated with collagen I or fibronectin after 4 days of entrapment. At least 12 cells analyzed per experiment; 3 independent experiments. Data are mean ± S.D. ^**^p < 0.01. Statistical comparisons are made using two-way ANOVA. (c) Schematic showing coating of a surface with CDH11-FC or CDH2-FC. (d) Quantification of CArG-RE activity on day 4 (normalized to day 0) in SMCs entrapped in 10- or 4-*μ*m microchannels coated with collagen I, CDH11-FC, or CDH2-FC. At least 12 cells analyzed per experiment; at least 3 independent experiments. Data are mean ± S.E.M. ^*^p < 0.05, ^**^ p < 0.01. Statistical comparisons are made using two-way ANOVA. (e) Percent decrease in SMC length in response to 0.25 *μ*M U-46619 in 10- or 4-*μ*m microchannels coated with collagen I, CDH11-FC, or CDH2-FC after 4 days of entrapment. At least 12 cells analyzed per experiment; at least 3 independent experiments. Data are mean ± S.D. ^*^p < 0.05, ^**^ p < 0.01. Statistical comparisons are made using two-way ANOVA.

Fibronectin, similar to collagen I, supported SMC proliferation in 10-*μ*m channels while reducing cell division in 4-*μ*m channels [Fig. 1(f) and supplementary material, Fig. 2(d)]. However, the reduction in SMC proliferation appeared more pronounced in tightly confined channels with collagen I compared to those with fibronectin [Fig. 1(f) and supplementary material, Fig. 2(d)]. Furthermore, in contrast to collagen I, fibronectin did not induce SRF-dependent gene expression in 4-*μ*m channels [Fig. 4(a)] as CArG-RE activation was found to be similar in 10-*μ*m and in 4-*μ*m channels coated with fibronectin [Fig. 4(a)]. Moreover, the enhanced contractile response to U-46619 observed in 4-*μ*m channels coated with collagen I was not replicated in channels photopatterned with fibronectin [Fig. 4(b)]. These data indicate that, unlike collagen I, fibronectin fails to promote the contractile phenotype in confined SMCs.

Cadherins mediate homophilic interactions between neighboring vSMCs.^44^ In particular, Cadherin-2 (CDH2) and Cadherin-11 (CDH11) have been shown to regulate SMC migration,^45,46^ proliferation^46,47^ and contractile function.^48^ To investigate how these cell adhesion molecules affected confined SMCs, we employed photopatterning to selectively coat the microchannel surfaces with either CDH2 or CDH11 fused with the human Fc region of IgG (CDH2-FC or CHD11-FC). The remaining device was treated with PLL-mPEG-SVA [Fig. 4(c)]. SMCs successfully entered these microchannels as cells could adhere to both CDH2-FC and CDH11-FC surfaces [supplementary material, Figs. 2(e) and 2(f)]. Interestingly, CDH11-FC, but not CDH2-FC, promoted CArG-RE activation in confinement [Fig. 4(d)]. This CDH11-induced upregulation of CArG-dependent gene expression manifested in both 4- and 10-μm channels and was similar in magnitude to the CArG-RE activation observed in 4-μm channels coated with collagen I [Fig. 4(d)]. Moreover, CDH11 engagement induced a robust contractile response to U-46619, which was independent of the degree of confinement [Fig. 4(e)]. In comparison, SMC contractility was lower in CDH2-FC-coated channels [Fig. 4(e)], resembling the response seen in 10-μm channels coated with collagen I. These findings demonstrate that CDH11 and CDH2 exert distinct effects on confined SMCs.

### SMC responses to TGF-β1 and compressive forces in confinement

**FIG. 5.**
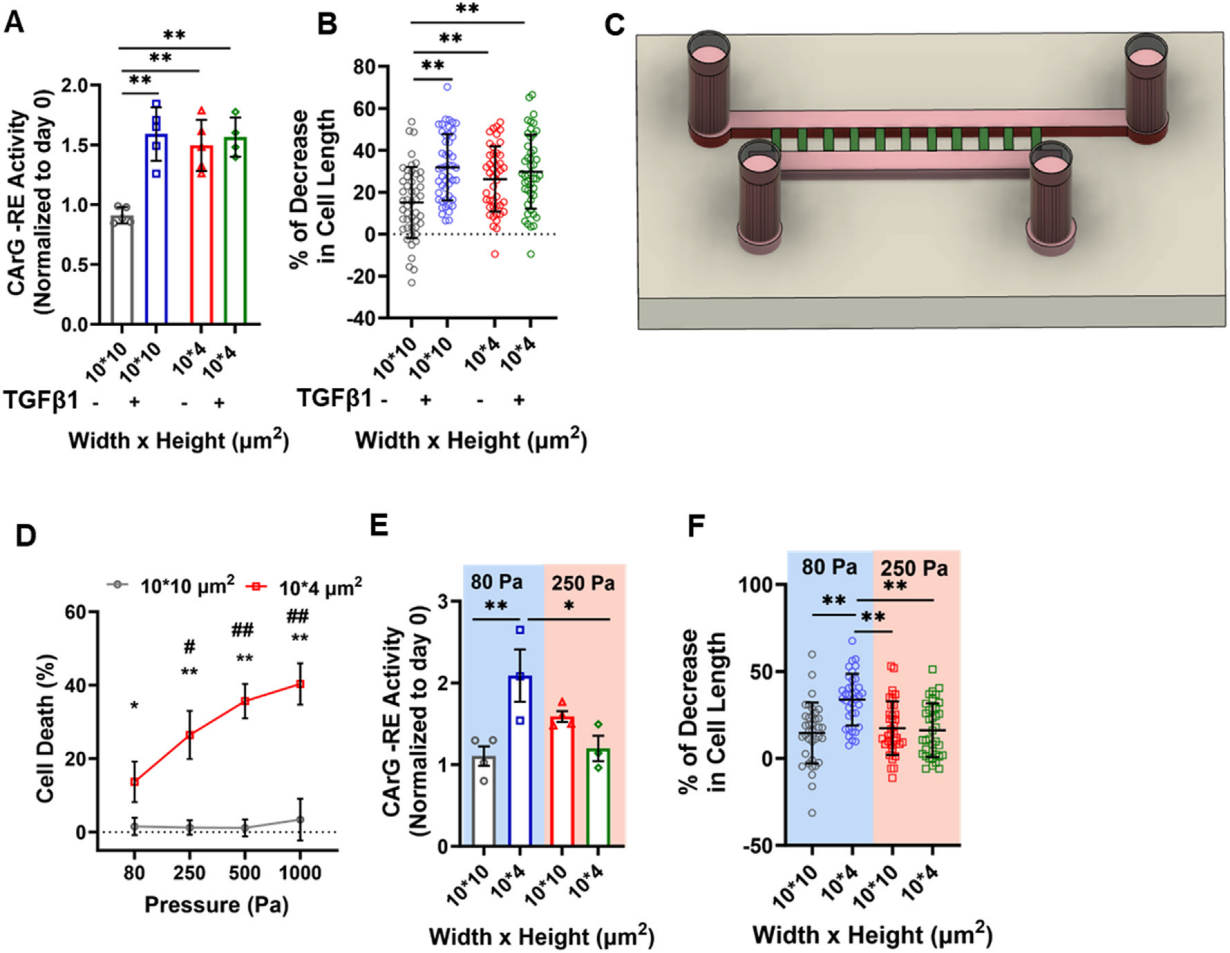
Mechanical and chemical cues regulate SMC gene expression and contractility in confinement. (a) Quantification of CArG-RE activity on day 4 (normalized to day 0) in SMCs entrapped in 10- or 4-*μ*m microchannels coated with collagen I in the presence or absence of TGF-β1 (2 ng/mL). At least 12 cells analyzed per experiment; at least 3 independent experiments. Data are mean ± S.E.M. ^**^ p < 0.01. Statistical comparisons are made using two-way ANOVA. (b) Percent decrease in SMC length in response to 0.25 *μ*M U-46619 in 10- or 4-*μ*m microchannels coated with collagen I. Prior to the contractility experiment, confined SMCs were cultured in the presence or absence of TGF-β1 (2 ng/mL) for 4 days. At least 12 cells analyzed per experiment; at least 3 independent experiments. Data are mean ± S.D. ^**^p < 0.01. Statistical comparisons are made using two-way ANOVA. (c) Schematic illustrating the application of pressure forces on confined SMCs. (d) Percentage of SMCs that die inside 10- or 4-*μ*m microchannels coated with collagen I in response to different levels of hydrostatic pressure. At least 50 cells analyzed per experiment; 4 independent experiments. Data are mean ± S.D. ^*^p < 0.05, ^**^p < 0.01 for comparison between 10- and 4-*μ*m channels. # p < 0.05, ## p < 0.01 relative to 80 Pa 4-*μ*m channels. Statistical comparisons are made using two-way ANOVA. (e) Quantification of CArG-RE activity on day 4 (normalized to day 0) in SMCs entrapped in 10- or 4-*μ*m microchannels coated with collagen I and cultured under 80 and 250 Pa pressure. At least 12 cells analyzed per experiment; at least 3 independent experiments. Data are mean ± S.E.M. ^*^p < 0.05, ^**^ p < 0.01. Statistical comparisons are made using two-way ANOVA. (f) Percent decrease in SMC length in response to 0.25 *μ*M U-46619 in 10- or 4-*μ*m microchannels coated with collagen I. Prior to the contractility experiment, confined SMCs were cultured under pressure of 80 or 250 Pa for 4 days. At least 12 cells analyzed per experiment; 3 independent experiments. Data are mean ± S.D. ^**^ p < 0.01. Statistical comparisons are made using two-way ANOVA.

TGF-β1 promotes the contractile phenotype of vSMCs [Figs. 2(a)–2(c)]. However, the impact of 3D confinement on SMC responses to TGF-β1 remains unknown. To this end, we cultured SMCs in 10- or 4-*μ*m channels in the presence or absence of this growth factor. For these experiments, we coated channels with collagen I to examine the potential additive effects of ΤGF-β1 and tight confinement on CArG-RE activation and contractility. We found that while treatment with TGF-β1 increased normalized CArG-RE activity in SMCs entrapped in 10-*μ*m channels, this growth factor failed to hyperactivate the CArG box in cells cultured in 4-*μ*m channels [Fig. 5(a)]. Along these lines, TGF-β1 enhanced the contractile function of moderately confined SMCs, without further augmenting contractility in tightly confined channels [Fig. 5(b)]. These data demonstrate that in the presence of collagen I, TGF-β1 induces the contractile phenotype in moderately confined SMCs, whereas combining TGF-β1 with a high degree of confinement fails to additively enhance SMC-specific gene expression and contractility.

In addition to chemical signals, vSMCs constantly experience compressive forces stemming from elevated blood pressure. To increase compressive forces in confinement, we elevated the hydrostatic pressure (HP) by increasing the medium height in the inlet and outlet wells [Fig. 5(c)]. Specifically, cells entrapped in collagen I-coated microchannels were exposed to HP levels ranging from 80 to 1000 Pa. Of note, all experiments presented in previous figures were conducted under an HP of 80 Pa. Our results showed that SMC viability decreased with increasing the magnitude of compressive forces [Fig. 5(d)]. This effect was observed solely in cells confined within 4-*μ*m channels, whereas those cultured in 10-*μ*m channels maintained nearly 100% viability [Fig. 5(d)]. Moreover, while the culture of SMCs in 4 *μ*m-channels coated with collagen I and under HP of 80 Pa activated CArG-RE [Fig. 5(e)], 250 Pa abolished this confinement-induced upregulation of CArG-RE activity [Fig. 5(e)]. 250 Pa also diminished SMC contractility in 4-μm channels to levels akin to those seen in 10-μm channels [Fig. 5(f)]. These findings indicate that tightly confined SMCs exposed to elevated compressive forces display low viability and diminished contractile function.

## DISCUSSION

*In vivo*, healthy arterial SMCs reside within confined environments and display a contractile phenotype. However, when isolated and cultured on planar surfaces, they transition to a synthetic and proliferative state.^49^ Although prior studies have explored the roles of chemical and mechanical stimuli in SMC phenotypic switching,^9,12^ the influence of 3D spatial restrictions in this process remains elusive. To address this, we designed a microfluidic device that provides precise control over the degree and duration of SMC confinement. Our assay allows for the investigation of SMC responses to ECM proteins, cell–cell adhesion molecules, growth factors, and compressive forces. Moreover, by quantifying changes in morphological parameters, such as cell length, area, and aspect ratio, we assess SMC contractility in response to the vasoconstrictors U-46619 and endothelin 1. Future integration of this assay with traction force microscopy^50^ and Brillouin microscopy^51^ could enable precise measurements of contractile forces and cellular stiffness in confined environments. A limitation of our assay is the use of the relatively stiff PDMS for fabricating the microchannels. Developing hydrogel-based devices with tunable elasticity would enable us to decouple the effects of substrate stiffness and confinement on SMC behavior.^50^

A vast body of research shows that the expression of nearly all contractile genes, including Myh11, desmin, SM22α, αSMA, smoothelin, and caldesmon depends on one or more CArG boxes.^34–39^ Activation of CArG-RE is also a marker of differentiation toward SMCs.^32,40^ In light of these results, we opted to measure CArG-RE activation using a live-cell reporter under various treatments and conditions rather than performing immunocytochemistry for individual contractile genes. This approach offers significant advantages: (a) CArG-RE activation reflects the upregulation of multiple contractile genes, rather than just one or two, which are often used to assess the presence of the contractile phenotype; (b) it provides real-time information on the kinetics of contractile gene activation, which is not achievable with end point assays like immunocytochemistry; and (c) it assesses the transcriptional activity of SRF, one of the key transcription factors orchestrating myogenesis.^9^ Importantly, using two distinct interventions, namely, TGF-β1 and SRF-VP16, both known to induce the contractile phenotype,^9,40–42^ we verified that the CArG-RE reporter performs as designed. Drawing on both the contractility and CArG-RE reporter experiments, we found that SMCs exhibit distinct phenotypic responses to biochemical signals, ECM proteins, and compressive forces in tight versus moderate confinement.

The ECM of the normal vascular media primarily consists of elastic fibers, fibril-forming collagens (types I, III, and V), fibronectin, and basement membrane components, including collagen IV, laminins, nidogens, and perlecan.^15^ Apart from providing structural support to blood vessels, the ECM plays a key role in inducing phenotypic switching. Previous research has shown that freshly isolated SMCs cultured on planar surfaces coated with collagen I acquire a synthetic phenotype.^52^ Moreover, both plasma fibronectin, which lacks extra domain A (EDA) and extra domain B (EDB), as well as the EDA-containing fibronectin isoform, which is typically found in the ECM, enhance SMC proliferation, migration, and expression of synthetic markers.^53–57^ Depletion of fibronectin-EDA in SMCs suppresses the SMC synthetic phenotype and decreases intimal hyperplasia and late atherogenesis in apolipoprotein E-deficient mice.^56,58^ In line with these results, we find that collagen I and plasma fibronectin support SMC proliferation and fail to upregulate contractile genes in moderately confined environments. Interestingly, under conditions of elevated confinement, collagen I, but not fibronectin, induces a contractile phenotype in SMCs as evidenced by the upregulation of αSMA and CArG-RE activity as well as the enhancement of the contractility in response to vasoconstrictors. Before being introduced into the device, SMCs were cultured on planar surfaces in a cell culture medium that promotes their proliferative and synthetic phenotype. Fibronectin appears to preserve this phenotype in confinement as it fails to activate CArG-RE.

Our data indicate that collagen I-coated, tightly confined channels increase the contractile response of adventitial fibroblasts to U-46619 suggesting that this microenvironment may also trigger their differentiation into myofibroblasts, which contribute to neointimal formation in animal models.^59,60^ Although the mechanisms driving the collagen I-induced acquisition of the contractile phenotype by SMCs and fibroblasts in tightly confined spaces are yet to be elucidated, our findings implicate the TGF-β pathway in this process. Using collagen I-coated microchannels, we demonstrate that while TGF-β1 treatment promotes the contractile phenotype in moderately confined SMCs, it fails to enhance CArG-RE activity and contractility in tightly confined cells, suggesting that the effects of tight confinement and collagen I on SMC phenotype are mediated through the TGF-β pathway. TGF-β uses both the canonical and non-canonical SMAD pathways to induce contractile gene expression.^61,62^ These pathways typically converge on three TGF-β responsive elements located in the promoter region of many SMC-specific genes: the TGF-β control element (TCE), the Smad binding element (SBE), and the CArG box.^41,42,63^ One of these non-canonical pathways that becomes hyperactivated in tight confinement is the RhoA signaling pathway.^64^ Activation of the RhoA pathway is sufficient to increase CArG-dependent gene expression and SMC contractility.^65–68^

Cadherins are cell surface molecules that mediate Ca^2+^-dependent homophilic cell–cell interactions.^69^ Cadherin engagement initiates signal transduction, influencing fundamental SMC processes such as cell polarity, migration, proliferation, survival, and differentiation.^44^ In this study, we focus on CDH2 and CDH11 due to their abundant expression in vSMCs.^44^ By decorating microchannels with CDH11-FC, we find that CDH11 engagement induces the contractile state of SMCs regardless of the degree of confinement. In contrast, homophilic engagement of CDH2 does not activate the CArG reporter and fails to enhance SMC contractility, mirroring the effects seen with fibronectin on confined SMCs. Our data align with previous research demonstrating that CDH11, but not CDH2, is essential for the derivation of contractile SMCs.^69^ CDH11 is also necessary for the *in vivo* development of contractile function, as shown using CDH11^−/−^ mice.^69^ Loss of CDH11 results in reduced expression of SMC proteins and attenuated contractile response of SMC-containing tissues, such as aortas and bladders, to different agonists.^69^ CDH11-induced SMC maturation depends on TGF-β receptor II and on the activation of SRF through the ROCK pathway, which promote SMC-specific gene expression.^69^ In agreement with this, we find that CDH11 triggers SRF-dependent gene expression in both 10- and 4-*μ*m channels.

Prior research has shown that exposure to increased confinement arrests cells in the S/G2/M phase of the cell cycle.^27^ Moreover, while prolonged confinement may initially lead to cell death, a fraction of cells eventually adapts and survives under these conditions.^24,70^ Evidence suggests that the underlying cause of both cell cycle arrest and cell death is confinement-induced DNA damage.^24,28,70,71^ This DNA damage arises from several mechanisms, including (a) increased oxidative stress caused by elevated intracellular reactive oxygen species;^28,70^ (b) nuclear depletion of DNA repair factors such as Ku70, Ku80, and BRCA1;^28,72^ and c) nuclear envelope rupture^72–74^ that leads to the translocation of the cytoplasmic exonuclease TREX1 into the nucleus.^75^ In line with previous work,^24,26–28^ we observed that tightly confined SMCs divide less frequently. Although a modest degree of SMC death is detected in tight confinement, the combination of elevated confinement and increased compressive forces suppresses CArG-RE activation and exacerbates confinement-induced cell death, presumably due to DNA damage surpassing the cell's repair capacity. DNA damage and the death of medial SMCs are key factors contributing to the progression of vascular disorders, such as atherosclerosis^76,77^ and aortic aneurysm.^3^ Moreover, pronounced nuclear envelope ruptures have been observed in aortic SMCs in a mouse model of Hutchinson–Gilford progeria syndrome (HGPS).^78^ These ruptures precede the loss of medial SMCs and likely contribute to the vascular pathology of HGPS, which shares similarities to vascular aging.^78,79^ More work is required to decipher the interplay between compressive forces and confinement in physiological and pathophysiological processes, including the development of vascular diseases.

## CONCLUSION

Our work provides a sophisticated, high-throughput microfluidic assay for investigating the process of SMC phenotypic switching in physiologically relevant confined environments. Importantly, we demonstrate that 3D confinement influences how SMCs respond to different chemical and physical stimuli. These findings underscore the significance of 3D spatial constraints in SMC biology.

## METHODS

### Cell culture, cell treatment with chemicals, lentiviral constructs, and lentivirus production

Human aortic smooth muscle cells were purchased from Lifeline Cell Technology. Cells were cultured in smooth muscle cell growth medium 2 (PromoCell) supplemented with 5% (v/v) growth medium (PromoCell) and 1% (v/v) penicillin/streptomycin (Gibco). This culture was kept at 37 °C and 5% CO_2_ and passaged every 2–4 days. All experiments were conducted using SMCs at passages 3–7. HAoAF were purchased from PromoCell and cultured in Fibroblasts growth medium 2 (PromoCell) supplemented with 2% (v/v) growth medium (PromoCell) and 1% (v/v) penicillin/streptomycin (Gibco). This culture was kept at 37 °C and 5% CO2 and passaged every 2–4 days. All experiments were conducted using HAoAF at passages 3–5. Experiments involving the agonist U-46619 (Enzo) were carried out at 0–5 *μ*M concentrations and experiments involving endothelin 1 (Millipore-Sigma) were carried out at 25 nM concentration. In select experiments, the cell culture medium was supplemented with TGF-β1 (2 ng/mL, USBiological). The CArG-RE LVDP vector (addgene plasmid #89762) and lentiviral constructs expressing only the Puro gene (CNT) or both SRF-VP16 and Puro were gifts from Stelios Andreadis.^40^ The control vector (CNT) contains the puromycin resistance gene (Puro) under the control of the constitutively active cytomegalovirus (CMV) promoter. The SRF-VP16 vector encodes both the SRF-VP16 gene and the Puro gene, driven by the same CMV promoter (CMV-SRF-VP16-IRES-Puro). Although puromycin selection could have been used to isolate a pure population of CNT and SRF-VP16 SMCs, we opted not to, as lentiviral particles efficiently transduce SMCs, with transduction efficiencies in our hands typically ranging from 80% to 95%. Of note, experiments using CNT and SRF-VP16 SMCs were carried out only a few days after transduction. Lentivirus was prepared as described previously.^24^ Lentiviral transduction was carried out in the presence of 8 *μ*g/mL polybrene (AmericanBio).

### Fabrication and photopatterning of microfluidic devices

We fabricated PDMS-based microchannel devices using standard multilayer photolithography and replica molding, as described previously.^51,64^ These devices had two larger, 2D-like channels and an array of parallel microchannels with constant length (200 *μ*m) and width (10 *μ*m) but variable heights (10-, 6-, 4-, or 3-*μ*m). The precise measurements for the 10-, 6-, 4-, and 3-*μ*m microchannels were as follows: 10-*μ*m channel (*W* × *H*) = 11.3 × 10.7 *μ*m^2^, 6-*μ*m channel (*W* × *H*)= 9.8 × 6.2 *μ*m^2^, 4-*μ*m channel (*W* × *H*) = 9.9 × 4.2 *μ*m^2^, and 3-*μ*m channel (*W* × *H*) = 9.8 × 3.1 *μ*m^2^, as assessed via a laser profilometer. To prepare these devices, PDMS prepolymer and crosslinker were mixed at a 10:1 ratio and poured onto a silicon wafer. After curing at 85 °C for 70 min, PDMS blocks were cut. Subsequently, coverslips and PDMS blocks were subjected to plasma cleaning for 1 min. Post plasma cleaning, the PDMS blocks were attached to coverslips, thereby creating the different microfluidic devices. For experiments involving staining of cells in microchannels (αSMA immunocytochemistry) only the coverslips went through the plasma cleaning process. The PDMS blocks were attached to glass coverslips and heated at 65 °C for 1 h to improve adherence. After this, the devices underwent a second plasma cleaning procedure. To selectively coat the different types of microchannels with the desired molecules, PRIMO photopatterning technology (Alveole) was employed. Devices were first incubated with 0.1% (v/v) PLL (Millipore-Sigma) for 1 h and washed with 10 mM HEPES buffer (pH = 8–8.5). Next, an anti-fouling layer was created on the devices by coating them overnight with 50 mg/mL mPEG-SVA (5kD; Laysan Bio). The anti-adhesive layer inside the microchannel walls was subsequently degraded by using the photoactivatable reagent PLPP (Alveole) and UV light. Microchannels were then coated with the following ECM molecules: rat tail collagen I (20 *μ*g/mL; Gibco), collagen I-FITC (20 *μ*g/mL; Millipore-Sigma), and fibronectin-FITC (40 *μ*g/mL; Millipore-Sigma) for 1 h at 37 °C, laminin (20 *μ*g/mL; SouthernBiotech) for 1 h at 37 °C and then overnight at 4 °C, and human plasma fibronectin (20 *μ*g/mL; Gibco) overnight at 4 °C.

For experiments investigating the effects of cell–cell adhesion molecules on SMC phenotypic switching, after treating the microchannels with PLPP and UV light, the devices were incubated with 10 *μ*g/mL goat anti-human IgG (FCy-specific, Jackson ImmunoResearch) in a Tris-buffered saline (TBS) solution (50 mM Tris-HCl [pH 8.0], 138 mM NaCl, 27 mM KCl) containing 1 mM CaCl_2_ for 18 h at 4 °C. Subsequently, devices were washed twice with binding buffer (10 mM HEPES [pH 7.4], 1 mM CaCl_2_, 137 mM NaCl, 5.4 mMKCl, 0.34 mM Na_2_HPO_4_, 0.1% glucose) and blocked with 1% bovine serum albumin (BSA; Millipore-Sigma) in binding buffer for 30 min at room temperature. After blocking, the devices were washed twice more with binding buffer, and then a solution of Human CDH11-FC (10 μg/mL; R & D Systems) or Human CDH2-FC (10 *μ*g/mL; R & D Systems) was added. The devices were incubated with this solution for 1 h at 37 °C. In select experiments, 2D glass bottom dishes were passivated using PLL and mPEG-SVA, then photopatterned with desired ECM/cadherin molecules as described above. Of note, photopatterning of microchannels leads to protein deposition on all surfaces within microchannels.^24^ Consequently, once cells enter the microchannels, they are able to adhere to all four walls.

### Cell seeding in microfluidic devices and live-cell imaging

To introduce cells in the seeding channel, 20 *μ*l of cell suspension (2 × 10^6^ cells/mL) was added to the inlet well. Then, cells were allowed to adhere adjacent to the microchannel entrances by incubating them for 10 min at 37 °C and 5% CO_2_. To facilitate SMC entry into microchannels, a chemotactic gradient was generated by filling the topmost 2D-like channel with smooth muscle cell growth medium 2 supplemented with 5% (v/v) growth medium and 1% (v/v) penicillin/streptomycin and the lowermost 2D-like channel with smooth muscle cell growth medium 2 supplemented with 1% (v/v) penicillin/streptomycin. Similarly, a chemotactic gradient was generated to induce HAoAF entry into microchannels by filling the topmost 2D-like channel with fibroblasts growth medium 2 supplemented with 2% (v/v) growth medium and 1% (v/v) penicillin/streptomycin and the lowermost 2D-like channel with fibroblasts growth medium 2 supplemented with 1% (v/v) penicillin/streptomycin. Following approximately 2–4 h of cell entry into microchannels, the chemotactic gradient media was removed and SMCs were cultured in either smooth muscle cell growth medium 2 supplemented with 5% (v/v) growth medium and 1% (v/v) penicillin/streptomycin, while HAoAF were cultured in fibroblast growth medium 2 supplemented with 2% (v/v) growth medium and 1% (v/v) penicillin/streptomycin. To prevent issues caused by evaporation of the cell culture medium, we performed media changes every 24 h. Cell imaging was conducted using a Nikon Ti2 Inverted Microscope equipped with a Tokai Stage-Top Incubator, enabling precise control of temperature, humidity, and CO_2_ levels. The cells were maintained at 5% CO_2_ and 37 °C during imaging. Fluorescent images were captured using TRITC, GFP, and DAPI filters. Additionally, a Nikon AXR confocal microscope was employed to visualize the deposition of collagen-FITC and fibronectin-FITC within the microchannels as well as to assess αSMA levels in confinement and on 2D surfaces.

### Quantification of cell entry, cell entrapment, cell division, cell migration, and cell death

The percentage of cell entry was calculated by dividing the total number of cells seeded next to microchannel entrances by the number of cells that fully entered the microchannels 2 h after applying the chemotactic gradient. The percentage of cell entrapment was calculated by dividing the number of cells that remained inside the microchannels by the number of cells that entered the microchannels. We excluded non-viable and dividing cells from the analysis. The percentage of cells that underwent division inside various microchannel configurations was quantified 18 h after channel entry. Time-lapse microscopy was employed to track cell division, distance traveled, and entry. The migration speed on day 4 of entrapment was determined by dividing the distance the cells traveled within the microchannel by the imaging duration (2 h). On the third day of cell entrapment, cell viability within the microchannels was assessed using live-dead staining (Biovision), following the manufacturer's guidelines. The number of live (green) and dead (red) cells was counted to determine cell viability. All analyses specifically focused on microchannels containing one cell.

### Quantification of CArG-RE

Fluorescent imaging was performed for 4 days (∼24 h intervals). ImageJ software (National Institute of Health) was used for image analysis. The normalized green-to-red fluorescence intensity (GFI/RFI) ratio was calculated for each cell inside microchannels at different time points using the following formula:

CArG-RE activity (normalized to day 0):

$$\frac{\left(\frac{{\text{GFI}}_{\text{Day}(x)}}{{\text{RFI}}_{\text{Day}0}}\right)}{\left(\frac{{\text{GFI}}_{\text{Day}0}}{{\text{RFI}}_{\text{Day}0}}\right)}=\frac{\left(\frac{{\left(\text{Integrated}\;\text{intensity}\;\text{GFI}\;of\;\text{the}\;\text{cell}-\text{Integrated}\;\text{intensity}\;\text{GFI}\;of\;\text{the}\;\text{background}\right)}_{\text{Day}(x)}}{{\left(\text{Integrated}\;\text{intensity}\;\text{RFI}\;of\;\text{the}\;\text{cell}-\text{Integrated}\;\text{intensity}\;\text{RFI}\;of\;\text{the}\;\text{background}\right)}_{\text{Day}0}}\right)}{\left(\frac{{\left(\text{Integrated}\;\text{intensity}\;\text{GFI}\;of\;\text{the}\;\text{cell}-\text{Integrated}\;\text{intensity GFI}\;of\;\text{the}\;\text{background}\right)}_{\text{Day}0}}{{\left(\text{Integrated}\;\text{intensity}\;\text{RFI}\;of\;\text{the}\;\text{cell}-\text{Integrated}\;\text{intensity}\;\text{RFI}\;of\;\text{the}\;\text{background}\right)}_{\text{Day}0}}\right)}.$$

### Contractility measurements

The U-46619 (Enzo) solution was prepared by diluting the drug in a cell culture medium to concentrations ranging from 0 to 5 *μ*M. The endothelin 1 solution was prepared by diluting the drug in a cell culture medium to a final concentration of 25 nM. Cells entrapped in microchannels of different dimensions were imaged before and after adding U-46619 or endothelin 1 over a period of 50 min. Image quantification was carried out using ImageJ. The percentage decrease in a morphological parameter (cell length, area, or aspect ratio) was calculated using the following formula:

$$\%\;\text{Decrease}\;\text{in}\;\;\text{a}\;\;\text{morphological}\;\text{parameter}=\frac{\text{morphological}\;\text{parameter}\;\left(\text{before}\;\text{drug}\;\text{addition}\right)-\text{morphological}\;\text{parameter}\;\left(\text{at}\;t=50\;\text{min}\;\text{after}\;\text{drug}\;\text{addition}\right)}{\text{morphological}\;\text{parameter}\;\left(\text{before}\;\text{drug}\;\text{addition}\right)}\times 100.$$

### SMC exposure to elevated compressive forces

To tune the magnitude of compressive forces acting on confined cells, we adjusted the height of the cell culture medium in the inlet and outlet wells. The gauge pressure was calculated using the following formula: P=ρgh, where ρ is the density of water (1000 kg/m^3^), g is the gravitational acceleration (9.81 m/s^2^), and h is the height of the cell culture medium (Table I).

**TABLE I.** Medium height in inlet and outlet wells and corresponding pressure.

h (cm)	0.8	2.55	5.1	10.2
P (Pa)	80	250	500	1000

### Immunocytochemistry against αSMA, YAP1, and Histone H3 and quantification

SMCs cultured in microchannels or on 2D surfaces were fixed for 10 min using 4% paraformaldehyde (Affymetrix Inc.), permeabilized for 15 min using 0.1% Triton X-100 (Millipore-Sigma), and blocked for 1 h using blocking buffer (1% bovine serum albumin (BSA) (Millipore-Sigma), 2% goat serum (Vector Laboratories), and 0.1% Triton X-100). Next, cells were incubated overnight with the αSMA antibody (1:100, Millipore-Sigma) or anti-YAP1 (1:50, Santa Cruz Biotechnology) together with anti-Histone H3 (1:400, Cell Signaling) followed by a 2-h incubation with Alexa Flour 568 goat anti-mouse (1:100, Thermo Fisher Scientific), 488 goat anti-rabbit (1:100, Thermo Fisher Scientific), and Hoechst 33342 (1:2500, Biotium). The primary and secondary antibodies were dissolved in the blocking buffer. Samples incubated only with Alexa Flour 568 goat anti-mouse/488 goat anti-rabbit (1:100, Thermo Fisher Scientific) and Hoechst 33342 served as negative controls. Prior to staining confined cells with αSMA, the devices were detached by inserting a razor blade between the PDMS block and glass coverslip.

Image analysis was performed using the ImageJ software. αSMA levels in individual SMCs were calculated using the following formula:

$$\text{Integrated}\;\text{intensity}\;\text{of}\;\alpha \text{SMA}=\text{Total}\;\text{intensity}\;\text{of}\;\alpha \text{SMA}\;\text{in}\;\text{cells}-\left(\text{Cell}\;\text{Area}\times \text{Mean}\;\text{Background}\;\text{Intensity}\right).$$

The YAP1 and Histone H3 nuclear-to-cytoplasmic ratio in individual SMCs was calculated using the following formula:

$$\frac{\text{Nuclear}}{\text{Cytoplasmic}}=\frac{\text{Mean}\;\text{fluorocence}\;\text{intensity}\;of\;\text{the}\;\text{nucleus}-\text{Mean}\;\text{fluorocence}\;\text{intensity}\;of\;\text{background}}{\text{Mean}\;\text{fluorocence}\;\text{intensity}\;of\;\text{the}\;\text{cytoplasm}-\text{Mean}\;\text{fluorocence}\;\text{intensity}\;of\;\text{background}}.$$

### Statistical analysis

All the experiments were performed at least 3 independent times. D'Agostino–Pearson omnibus and Anderson–Darling normality tests were used to assess if the data followed a normal distribution. The following statistical tests were used to determine statistical significance (p < 0.05): Unpaired t*-*test, one-way analysis of variance (ANOVA) test followed by a Tukey's multiple comparisons test and two-way ANOVA followed by Tukey's multiple comparisons test. The ROUT method was employed to identify outliers. Analysis was performed using GraphPad Prism 10 software.

## SUPPLEMENTARY MATERIAL

See the supplementary material for all supplemental figures cited in the manuscript.

## Data Availability

The data that support the findings of this study are available from the corresponding author upon reasonable request.
